# High Resolution Based Quantitative Determination of Methylation Status of *CDH1* and *VIM* Gene in Epithelial Ovarian Cancer

**DOI:** 10.31557/APJCP.2019.20.10.2923

**Published:** 2019

**Authors:** Gaurav Kr Thakur, Tusha Sharma, T Krishna Latha, B D Banerjee, Harendra Kr Shah, Kiran Guleria

**Affiliations:** 1 *Environmental and Molecular Biology Laboratory, Department of Biochemistry,*; 2 *Department of Obst and Gynae, University College of Medical Sciences (Delhi University) and GTB Hospital, Dilshad Garden, Delhi, India. *

**Keywords:** Methylation, epithelial ovarian cancer, HRM, biomarker

## Abstract

**Background:**

DNA promoter methylation is widely explored epigenetic phenomena, known to effect gene expression and further perturbation in cellular homeostasis. Myriad of studies have leveraged promoter methylation and its potential as biomarker for various types of cancer. Aim of present study is to investigate promoter methylation of *CDH1* and *VIM* gene and etiology of epithelial ovarian cancer (EOC).

**Methods:**

Most of previous studies were qualitative; we have quantitatively assessed methylation levels in 50 EOC cases and control each through high recognition melt (HRM) technique.

**Results:**

At 10 % cutoff for *CDH1* 94% of EOC cases were found to be methylated with mean methylation of 45±13.8, whereas for control mean methylation was found to be 7.3±3.7 amongst 16 % methylation positive control samples. For *VIM* methylation was detected in 96% of cases with mean of 50.44±11.7 in EOC and in 12% methylation positive samples for control mean methylation was 6.24±4.3.

**Conclusion:**

In short HRM based DNA methylation can serve as a robust and sensitive diagnostic method for promoter methylation detection and as a biomarker for early epithelial ovarian cancer detection.

## Introduction

Among gynecological malignancies, ovarian cancer is 7th most common cancer worldwide and 8th leading cause of morbidity among women globally, of which Epithelial ovarian cancer (EOC) accounts for approx 90 % of all cases (Ferlay et al., 2015). At early stages symptoms associated with it are ambiguous and asymptomatic. Any sensitive screening test is lacking at moment, resulting in late diagnosis. As patients are diagnosed at later or advanced stages where disease has propagated and disseminated within peritoneal cavity rendering it nearly impossible for complete debulking. Late diagnosis results in very dismal survival which is nearly 30%, whereas survival chance improves to 90% if detected at earlier stages (Barton et al., 2008). Hence necessitating exploration of new avenues to brighten survival of EOC patients is required to be investigated.

Delineating molecular mechanisms that orchestrate pathogenesis is imperative and crucial for finding biomarkers. A multitude of genetic aberrations is responsible for evoking EOC but recent studies of epigenetic paradigm have begun to be appreciated (Barton et al., 2008; Balch et al., 2009; Khan et al., 2015). Alteration in methylation is crucial epigenetic mechanism and is key driver behind onset of cancer (Esteller and Herman, 2002; Laird, 2003; Pouliot et al., 2015). Aberrant events of methylation are key alternative to mimic role of tumor suppressor gene (TSG) mediated silencing (Baylin and Herman, 2000; Warnecke and Bestor, 2000; Jones and Baylin, 2002; Herman and Baylin, 2003).

Methylation-specific high recognition melt (MS-HRM) has emerged as a powerful technique for quantification and validation of methylation signatures associated with genes (Reed et al., 2007; Wojdacz and Dobrovic, 2007; Kristensen et al., 2008). We have focused on investigating *CDH1* and *VIM* gene among plethora of genes responsible for onset of EOC (Rathi et al., 2002; Teodoridis et al., 2005) . E Cadherin’s role as tumor suppressor is well investigated and its deregulation gears the journey of cells towards metastasis and EMT (Cowden Dahl et al., 2008; Onder et al., 2008; Zheng et al., 2009). Methylation of E cadherin promoter has found to be associated with advent of EOC (Bhagat et al., 2013). The up-regulation of vimentin (*VIM*) which has mesenchymal traits, induces metastasis and reinforce EMT phenomenon (Yang and Weinberg, 2008). Aberrant methylation of *VIM* gene has been reported earlier in several carcinomas including ovarian, colon, colorectal, bladder, etc (Chen et al., 2005; Costa et al., 2010; Zhou et al., 2014) .

In the Pursuit of exploring methylation-based biomarkers for EOC, we have focused to quantify promoter methylation of *CDH1* and *VIM* gene using HRM as a technique, which offers a robust, sensitive and cost-effective method for quantification of *CDH1* and *VIM* gene, methylation status, in EOC patients and matched ovarian tissue.

## Materials and Methods

*Tissue sample Collection*

A total of 100 tissue samples were collected comprising 50 each of EOC cases and normal control samples. Histopathologically proven EOC Samples were obtained from the patients undergoing surgery at Obst and Gynae department, GTB Hospital, Delhi. Normal ovarian tissue samples were obtained from patients undergoing hysterectomy for reason other than carcinoma. Those patients who had received treatment earlier for EOC had presence of secondary tumor, and any other histopathological types of ovarian cancer were excluded from the study. Patient informed consent was obtained from those patients who participated in this study, complying with institutional ethical guidelines.

*Control DNA*

Bisulphite converted universal methylated human DNA standard (D5014), Zymo research corp (USA) was used as 100% methylated control. DNA isolated from peripheral blood leucocytes were used as unmethylated control. Methylated and unmethylated controls were mixed in different ratios to give methylated standards of 0%, 10%, 25%, 50%,75% and 100%. These methylated standards were used for the preparation of standard methylation curve and regression analysis for quantifying methylation status.

*DNA Extraction and Bisulphite modification*

DNA was extracted from the tissues(25mg) using Quick g-DNA Miniprep (Zymo Research Corp, USA), in accordance with manufacturer instructions. Extracted DNA was subsequently quantified on Nanodrop 2000 (Thermo Scientific, USA) and stored in -20˚C till further use. 

Extracted DNA (500 ng) was bisulfite modified using EZ methylation Gold Kit ( Zymo Research Corp, Orange, CA). 130 µl of CT conversion Reagent was added to 20 µl of DNA sample (500ng) and was incubated at 98˚C for 10 minutes, followed up with incubation at 64˚C for 2.5 hrs. The converted DNA was then column purified and desulphonated, in accordance with manufacturer instructions. DNA was finally eluted in 10ul elution buffer. Upon bisulfite modification, all non-CpG cytosines got deaminated and converted into uracil, whereas methylated cytosine remained protected and maintained its methylation imprint.

**Figure 1:**
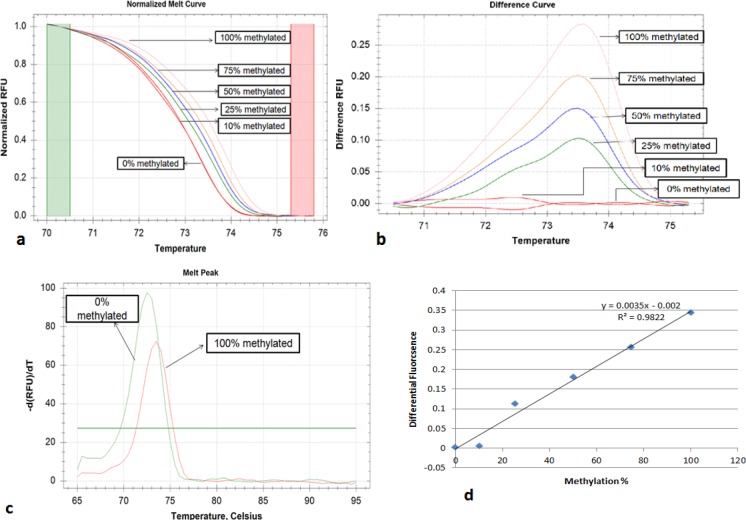
Representing Melt Plots of E-Cadherin. a, Normalized melt curve; b, Differential melt curve; c, Melt curve of 100% & 0% methylated; d, Regression Plot of Differential fluorescence vs methylation%.

**Figure 2:**
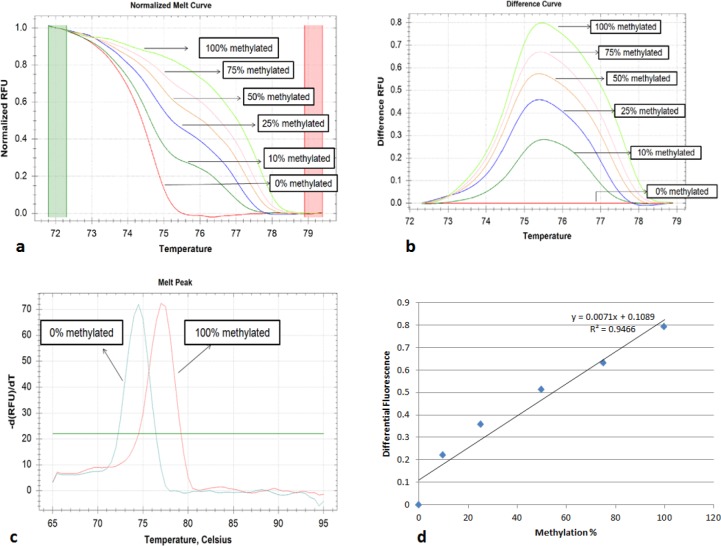
Representing Melt Plots of E Vimentin. a, Normalized melt curve; b, Differential melt curve; c, Melt curve of 100% & 0% methylated; d, Regression Plot of Differential fluorescence vs methylation%.

High Resolution melt analysis:- MS-HRM reaction was performed on CFX connect real-time PCR (Bio-Rad, USA) and melt profiles were further analyzed with precision melt software (Bio-Rad, USA). Cognate MIP primers were designed using methprimer software (Li lab, USA), to amplify target region regardless of methylation status (Wojdacz and Dobrovic, 2007). Primer sequence, product length, Tm value are given in (Table 2). PCR reaction was set up for 20 µl volume, containing 10 µl of precision melt supermix (Bio-Rad, USA), 1 µl each of 10µM forward and reverse primers, 2 µl bisulfite converted DNA, and rest of volume was made up with nuclease-free water. The amplification consisted of 5 min at 95˚C, and 40 cycles of denaturation for 10S at 95˚C, appropriate annealing temperature, and extension for 20S at 72˚C, following which melt analysis was done, between temperature 65˚C to 90˚C with ramping rate of 0.2˚C/5s increase in temperature per cycle, following which fluorescence signal was recorded. Precision melt software allowed us to normalize melting curve before and after fluorescence decrease (Figure 1a, 2a). A differential fluorescence plot was obtained after unmethylated control was set as baseline and other fluorescence was normalized against it (Figures 1b and 2b). Further a normalized melt curve of 100% methylated vs 0% was also obtained (Figure 1c and 2c).

*Melt Profile Analysis*

The premise of HRM based methylation quantification is based on the fact that upon bisulfite modification all non-CpG cytosine get converted to uracil, resulting in drop in GC content, which gets reflected in decreased Tm value. Different Tm values gave different RFU profiles in melt analysis. When we plotted normalized differential RFU of methylated standards (Tables 3 and 4) taking unmethylated control as baseline against methylation percentage we got a standard regression curve which was used for determination of methylation status of unknown samples for *CDH1* (Figure 1d and 2d).

*Statistical Analysis*

Data was analyzed using IBM SPSS16 version. Statistical significance for methylation status among EOC samples and controls was performed using independent sample t-test through comparison between mean methylation levels. Binary logistic regression was used to find risk of promoter methylation and etiology of epithelial ovarian cancer

## Results

Patients Characteristics: The mean age of EOC patients were 53.8±8.4 years, significantly higher than control group 50±6.85 years (P≤0.00). On basis of histopathology cases were categorized in 5 subtypes of which, 31 was of serous subtype, 06 were of mucinous subtype, 3 endometroid, 04 clear cell and 6 cases were of borderline origin. The Comparison of socio-demographic characteristics between case and control are provided in Table 1.

**Table 1:** Comparison of Various Socio- Demographic Features in Two Groups

Characteristics	Cases (n==50)	Control (n==50)	P value
Age (in years)	53.8± 8.4	50±6.85	0.00*
Residential Area			
Urban	47 (94%)	50 (100%)	0.760
Rural	3 (6%)	0 (0%)	
Socio Economic Status			
Lower	0 (0%)	0 (0%)	0.637
Middle	40 (80%)	43 (84%)	
Higher	10 (20%)	07 (14%)	
Occupation			
Unemployed	50 (100%)	45 (90%)	0.540
Employed	0 (0%)	5( 10%)	
Education			
Illiterate	33 (66%)	39 (78%)	0.432
Literate	17 (34%)	11 (22%)	
Dietary Habit			
Vegetarian	34 (68%)	28 (56%)	0.638
Non-vegetarian	16 (32%)	22 (44%)	
BMI( Kg/ m^2^)			
Underweight(< 18.5)	6 (12%)	0.290	
Normal ( 18.5-22.9)	15 (30%)	11 (22%)	
Overweight(23-24.9)	7 (14%)	8 (16%)	
Obese (>25)	22 (44%)	31 (62%)	
Use of Tobacco			
Yes	0 (0%)	0 (0%)	NA
No	50 (0%)	50 (100%)	

**Table 2:** Primer Sequence

Target	Primer Sequence	Amplicon Size	Annealing Temp (˚C)	No. of CpG in product
E Cadherin (CDH1)	GGTTGGGTAATATAGGGAGATATAG(F)	122	58	4
	TAAAAATACAAATACACACCACCAC(R)			
Vimentin (VIM)	ATTTTTTTAGAAAGGTTAAGGTGAT(F)	186	56	5
	CAACAATACACAATACAAAATTCAC(R)			

**Table 3:** RFU vs Methylation for E- Cadherin

Methylation Percentage	Differential RFU
100	0.344
75	0.255
50	0.179
25	0.114
10	0.005
0	0

**Table 4:** RFU vs Methylation for Vimentin

Methylation Percentage	Differential RFU
100	0.798
75	0.629
50	0.511
25	0.356
10	0.222
0	0

*Status of CDH1 and VIM Promoter Methylation*

We have applied HRM technique for promoter methylation of *CDH1* and *VIM* gene promoter region. Putting the value of differential fluorescence in regression formula, from diluted methylated standards, percentage methylation among unknown samples was obtained. Cut off value was set 10% methylation value, as we were able to detect 10% DNA methylation in background of unmethylated DNA, as reported earlier also (Meng et al., 2012). At 10% cutoff, methylation was detected in 47 out of 50 cases (94%) for *CDH1* with mean methylation level of 45±13.8. Among control group, methylation was detected in 8 samples with mean methylation around 7.3±3.7. The percentage of promoter methylation was significantly higher in cases than those of controls (P<0.001). For *VIM* gene methylation status, in 48 (96%) samples methylation was detected above 10% cutoff for cases, whereas only 6 (12%) samples showed traces of methylation in control group. Further mean methylation was found to be 50.44±11.7 in cases which were significantly higher than control subjects (P<0.001) showing mean methylation around 6.24±4.3. 

Assessing risk of EOC associated with Promoter methylation: Assessing risk while calculating odds ratio, it was found that risk of developing EOC was 5.8 times among cases indicating significantly higher than control in case of *CDH1* promoter methylation (P<0.001) whereas propensity to develop EOC was significantly (P<0.001) 8 time in cases as compared to controls for *VIM* gene promoter methylation.

## Discussion

Late diagnosis of epithelial ovarian carcinoma (EOC) at advanced stages is a major deterrent behind better and successful targeted medical care, resulting in grim 5-year survival rate. Early detection of onset of EOC might come as shot in arm, which could provide ample time for targeted therapy to nip the EOC in bud itself. 

Methylation based epigenetic modifications are widely studied the mechanism behind onset of many cancers. Methylation events are initial signatures, before any normal cellular machinery coming to halt and transforming cells towards cancerous state. Hence promoter methylation can be leveraged as an excellent prognostic biomarker for epithelial ovarian cancer. Promoter methylation has been studied earlier in epithelial ovarian cancer (Makarla et al., 2005) but in most of papers either MSP or bisulfite sequencing was used for promoter methylation analysis (Rathi et al., 2002; Bhagat et al., 2013). The bisulfite sequencing remains the gold standard in promoter methylation studies, but as all labs don’t have access to sequencers HRM has afforded a robust alternative. The purpose of study was to check robustness of HRM as a sensitive diagnostic tool for early detection of epithelial ovarian cancer as pioneered by Wojdacz and Dobrovic, (2007). In this study we have endeavored HRM based quantification, which has been utilized earlier for methylation detection for other cance (Kristensen and Hansen, 2009; Licchesi and Herman, 2009). Methylation analysis of *CDH1* and *VIM* gene in EOC was detected in which melting pattern of amplicons was compared for methylation quantification, providing a potentially simple, sensitive, reproducible and cost-effective methodology for EOC screening. 

In this study, we found differential methylation profile of EOC samples and controls, where level of methylation tends to be significantly higher in cases as compared to control. Further, significant age difference was reported between case and control. The reason can be attributed to fact that average age of women who present themselves for diagnosis for epithelial ovarian cancer (EOC) worldwide and in Indian scenario falls in higher age bracket as reported by Murthy et al., (2009). Further, symptoms associated with epithelial ovarian cancer get revealed mostly in late stages, resulting in higher age of the cases. As mean age was found to be significant in EOC cases, higher methylation in EOC cases is incidental to age. *CDH1* gene which encodes for a transmembrane glycoprotein mediates homophilic cell to cell interaction and adhesion maintaining cellular integrity. All the previous studies regarding promoter methylation of *CDH1* in EOC were studied through MSP as meta-analyzed by Wang et al., (2016) where *CDH1* promoter methylation ranged between 21% to 64% for cases whereas for control it hovered between meager 2.3% to 25 %. This study detected methylation in 94% of samples in cases whereas out of 50 controls methylation was detected in 8 samples with mean methylation of around 45% for cases and 7.3% for the controls, which is consistent with previous studies. The risk of developing EOC was found to be more in *CDH1* promoter methylation (OR 5.8) as compared to nonmalignant controls. Hyper-methylation of *VIM* gene has been studied and quantified in several previous studies (Kitamura et al., 2009; Shirahata et al., 2009). *VIM* gene encodes for Vimentin a member of intermediate filament family. It performs a pivotal role in maintaining cellular architecture and tissue integrity. *VIM* is considered as an important marker of epithelial-mesenchymal transition where cells acquire increased motility and invasiveness (Ivaska et al., 2007). Hypermethylation of *VIM* was thought to suppress vimentin expression but in most of cancer vimentin levels were overexpressed which appears paradoxical indicating *VIM* hypermethylation may involve in oncogenic trait. Further erratic promoter methylation of *VIM* gene has been implicated in various epithelial cancers. According to previous studies, there was very low level of methylation in normal colon cells as compared to colon cancer cells, wherein CpG region significantly elevated the level of methylation was found (Chen et al., 2005). Moreover *VIM* gene was found to be heavily methylated in well-differentiated gastric adenocarcinoma (Kitamura et al., 2009). In cervical cancer, previous studies have shown aberrant increased methylation profile (Jung et al., 2011; Lee et al., 2014). In primary colorectal carcinoma samples high methylation was reported in cases as compared to control (Shirahata et al., 2009). Most of previous studies employed qualitative MSP as detection method of methylation study, which is quite error-prone and there are chances of false positives. Our study investigated role of *VIM* promoter methylation in EOC progression through quantitative melting analysis. We found mean methylation level of *VIM* promoter region around 50.4% which was quite significant to normal ovarian samples. Further MS-HRM detected 48 (96%) methylation positive samples among EOC cases whereas there were only 6 (12%) with methylation of 6% which was above 10 % threshold, it was found there was increased risk of *VIM* promoter hypermethylation developing EOC (OR 8.0). This is the first study to the best of our knowledge which had studied impact of promoter methylation of *CDH1* and *VIM* gene in epithelial ovarian carcinoma and employed HRM for methylation quantification, it is one of strength of the present study. Further studies are needed to be carried out with panel of other biomarkers to establish efficacy of HRM as a quantification technique for methylation detection. As this work was conducted as a case-control study all cases of Epithelial Ovarian cancer were included in study. The intragroup study of various subtypes of EOC was not envisaged. Further as incidence of serous cancer is close to 75% among all the EOC cases, our results are consistent with previous studies as reported in previous study (Reid et al., 2017). Further, the correlation between methylation status and tumor subtypes of EOC and their metastatic potential can be planned as a sequel to this study.

In conclusion, we have demonstrated HRM as a sensitive and robust technique for detection of promoter methylation in epithelial ovarian cancer. Further high level of methylation levels were detected in *CDH1* and *VIM* promoter. HRM offers an easy and reliable potential screening method for EOC. Further studies are warranted to validate MS-HRM as a viable prognostic technique and its clinical importance.
